# The talin1-p53 axis inhibits osteocyte senescence to promote bone mass and mediate skeletal adaptation to mechanical stimulation

**DOI:** 10.7150/thno.123532

**Published:** 2026-01-01

**Authors:** Jianmei Huang, Lei Qin, Yishu Wang, Qinnan Yan, Haonan Yang, Haojin Chen, Mingyue Wu, Weihong Yi, Jiaming Yang, Sixiong Lin, Weiyuan Gong, Peijun Zhang, Shuangshuang He, Xinzhou Zhang, Guozhi Xiao

**Affiliations:** 1 Department of Biochemistry, Homeostatic Medicine Institute, School of Medicine, Guangdong Provincial Key Laboratory of Cell Microenvironment and Disease Research, Shenzhen Key Laboratory of Cell Microenvironment, Southern University of Science and Technology, Shenzhen, 518055, China.; 2 Department of Nephrology, The First Affiliated Hospital, Southern University of Science and Technology, Shenzhen People's Hospital, Shenzhen 518020, China.; 3 Orthopaedic Innovation Laboratory, Department of Orthopaedics, Shenzhen Nanshan People's Hospital, Affiliated Nanshan Hospital of Shenzhen University, Shenzhen, 518052, China.; 4 The Tenth Affiliated Hospital, Southern Medical University, Dongguan People's Hospital, Dongguan, 523059, China.; 5 School of Medicine, Southern University of Science and Technology, Shenzhen, 518055, China.; 6 Department of Orthopaedics, The First Affiliated Hospital of Guangzhou Medical University, Guangzhou, 510120, China.

**Keywords:** Talin1, p53, osteocyte senescence, bone homeostasis, force adaptation

## Abstract

**Background:** Osteoporosis is a major public health concern worldwide. As the predominant and long-lived bone cells, osteocytes serve as key regulators of bone remodeling and mechanotransduction. However, the molecular mechanisms underlying their regulatory roles remain poorly understood. The roles of talin1, a key focal adhesion protein linking integrins to the cytoskeleton, in regulation of osteocyte function and skeletal homeostasis remain unclear.

**Methods:** Osteocyte-specific talin1 conditional knockout (cKO) mice were established, and their skeletal phenotypes were assessed through micro-CT, histomorphometry, and biomechanical analyses. Osteocyte senescence and molecular signaling were assessed by RNA sequencing analysis, immunostaining, and biochemical assays. Talin1-p53 interactions were characterized by co-immunoprecipitation and pull-down assay. Rescue experiments were performed using talin1 and p53 double KO mice.

**Results:** Talin1 expression in osteocytes was markedly reduced during skeletal aging in mice and humans. Osteocyte-specific deletion of talin1 disrupted FA integrity and dendritic networks, leading to severe osteopenia in weight-bearing bones and impaired bone mechanical properties. Talin1 deficiency altered the bone marrow microenvironment, suppressing osteoblast differentiation while enhancing adipogenesis. Mechanistically, talin1 bound and sequestered p53 in the cytoplasm for proteasomal degradation. Thus, talin1 loss enhanced p53 nucleotranslocation, inducing upregulation of p16 and p21 and osteocyte senescence. Importantly, genetic ablation of p53 in osteocytes rescued the low bone mass phenotype, defective bone formation, and excessive senescence caused by talin1 loss.

**Conclusions:** This study identifies talin1 as a key factor governing osteocyte senescence and bone mass. We define a novel talin1-p53 axis that links impaired focal adhesion signaling to osteocyte senescence and bone loss, highlighting potential therapeutic targets for aging-related osteoporosis.

## Introduction

Cells rely on their ability to perceive and adapt to external stimuli, a process central to physiological activities and pathological conditions like migration, polarization, differentiation, and cancer dissemination 1-3. This process largely relies on focal adhesions (FAs), which are large macromolecular complexes linking the extracellular matrix (ECM) to the cytoskeleton. These complexes are formed by transmembrane integrins, the intracellular cytoskeleton, and other FA-associated proteins 4, 5. Talin has emerged as a key FA-associated protein that activates integrins and mediates force transmission between cytoskeleton and cell adhesions 6, 7. Talin also serves as an adaptor protein, recruiting numerous FA proteins and signaling molecules to adhesion sites and coordinating the stabilization of actin and microtubules during the adhesion process 3, 5. Previous studies have highlighted the critical roles of talin in tumor development and metastasis 8, 9 .

In vertebrates, talin exists as two isoforms, talin1 and talin2, which share 76% sequence identity and 88% similarity, yet display distinct tissue-specific expression patterns and functional roles during development 5, 10. Mice with global *talin1* knockout are embryonically lethal 11, whereas mice with *talin2* deletion are viable and fertile, presenting a mildly dystrophic muscle phenotype 12. Talin1 is ubiquitously expressed in nearly all cell types and tissues, while talin2 expression varies in different tissues with the high protein levels found in the cerebral cortex, heart, muscle and kidneys 13. Thus, large attention has been put to decipher the specific functions of talin1 in different tissue background. Recent studies have implicated that talin1 is involved in pathogenesis of human diseases, such as systemic capillary leak syndrome 14 and coronary artery dissection 15. Clinical evidence suggest a potential contribution of talin1 during the progress of skin fibrosis 16, coronary artery disease 17 and neurodegenerative disease 18. Mouse genetic studies have demonstrated the important roles of talin1 in regulating the formation and function of organs, including kidney, pancreas and heart 19-21. However, the function and importance of talin1 in skeletal homeostasis and function are largely unknown.

Vertebrates undergo constant bone remodeling by tightly controlled coupling of the osteoclast-mediated bone absorption with the osteoblast-mediated bone formation 22, 23. Osteocytes, the most abundant and long-lived bone cells, act as central regulators of bone remodeling, coordinating its initiation, modulation, and termination through direct and indirect interactions with osteoblasts and osteoclasts 24, 25. Accumulating evidence from our group and others indicates that several focal adhesion-associated proteins in osteocytes, including kindlin-2 26, 27, pinch1/2 28, integrin β1/3 29, 30 and FAK 31, are essential for proper bone remodeling. Genetic ablation of genes encoding these proteins in osteocytes led to cell dysfunction, abnormal bone remodeling, low bone mass, and defective force adaptation. However, molecular mechanisms whereby FA proteins regulate osteocyte function and bone remodeling are still largely unknown.

In this study, we demonstrate that talin1 is highly expressed in osteocytes in young and adult mice and humans and down-regulated during skeletal aging. The loss of talin1 in osteocytes causes a striking bone loss in both young adult and aged mice via up-regulation of transcription factor p53 and promotion of osteocyte senescence. Talin1 loss largely impairs mechanical stimulation responses in osteocytes and in bone. Talin1 ablation causes abnormal bone remodeling and alters MSC differentiation fate favoring adipogenesis versus osteogenesis in the bone microenvironment. p53 ablation in osteocytes prevents the bone loss in talin1-deficeint mice. We demonstrate a critical role of the talin1-p53 axis in osteocytes in modulation of bone mass and skeletal anabolic response to mechanical stimulation.

## Materials and Methods

### Ethics statement

All experimental related to animals were authorized by the IACUC of the Southern University of Science and Technology (SUSTech-JY202202068-202503A1). Human bone samples (Additional case details are provided in Table S1) were collected after surgery from the Shenzhen Nanshan People's Hospital. The study has been approved by the medical ethics regulations of Shenzhen Nanshan People's Hospital, China (No. KY-2021-053-01). Written consent was secured from each patient, and specimens were either directly used for protein extraction or fixed in 4% formaldehyde for IHC/IF staining.

### Animal studies

*Dmp1-Cre* mice were described previously 26, and *talin1^fl/fl^* and *p53^fl/fl^* mice were obtained from Shanghai Model Organisms. Experimental mice were generated using a two-step breeding strategy. Homozygous *talin1^fl/fl^* mice were initially crossed with* Dmp1-Cre* transgenic mice to generate *Dmp1-Cre; talin1^fl/+^* offspring. These heterozygous mice were then intercrossed with *talin1*^fl/fl^ mice to obtain *Dmp1-Cre; talin1^fl/fl^* mice. To generate *Dmp1-Cre*; *talin1^fl/fl^*;* p53^fl/fl^* mice, *Dmp1-Cre*;* talin1^fl/fl^* mice were first crossed with *p53^fl/fl^* mice to produce *Dmp1-Cre*; *talin1^fl/+^*;* p53^fl/+^* offspring, which were then intercrossed with *talin1^fl/fl^*;* p53^fl/fl^* mice to produce the *Dmp1-Cre*; *talin1^fl/fl^*; *p53^fl/fl^* mice and other desired genotypes. In vivo tibial loading was applied to the mice according to the established method 27, 32 to assess bone adaptation. Primer sequences for related genotyping are listed in Table S2.

### Micro-computerized tomography (μCT) analysis

Fixed, non-demineralized specimens of the femur, spine, tibia and skull were analyzed using micro-computed tomography (μCT) at the Experimental Animal Center of Southern University of Science and Technology. Scanning was performed using a Bruker μCT system (SkyScan 1176, Bruker MicroCT, Kontich, Belgium). All μCT acquisitions and subsequent image analyses were conducted in accordance with the standardized techniques, terminology, and reporting guidelines recommended by the American Society for Bone and Mineral Research (ASBMR) to ensure reproducibility and comparability of bone structural measurements 33.

### Histological evaluation and bone histomorphometry

Bone tissues were fixed in 4% paraformaldehyde (PFA) at 4 °C overnight and subsequently decalcified in 12% EDTA for 3 weeks. After sequential dehydration, bone tissues were embedded in paraffin and sectioned at the thickness of 5-μm used for hematoxylin and eosin (H&E) and tartrate-resistant acid phosphatase (TRAP) staining following the standard protocols as described previously 28, 34.

### Immunohistochemistry (IHC) and immunofluorescence (IF) staining

For IHC, 5-μm-thick bone sections were incubated with primary antibodies or control IgG and visualized using the EnVision+ System-HRP (DAB) kit (Dako North America Inc., Carpinteria, CA, USA) following previously established protocols 26, 35. For IF staining, both bone sections and MLO-Y4 cells were processed and labeled with specific antibodies according to methods described previously 36. Fluorescent signals were detected using a confocal microscope, and images were analyzed to evaluate protein localization and expression patterns.

### BMSC culture

Primary BMSCs were isolated from the femurs and tibiae of 6-month-old male mice following previously established protocols 26. Cells were cultured in 10-cm dishes containing α-MEM (Hyclone, USA) supplemented with 15% FBS at 37 °C in a 5% CO₂ incubator. After 24 h, non-adherent cells were collected by centrifugation (1500 rpm, 5 min), resuspended in 100 μl proliferation medium (base medium supplemented with 10 μg/ml M-CSF), and seeded into 96-well plates at a density of 2 × 10⁵ cells per well for BMM induction assays, as previously described 37. Adherent BMSCs were maintained for one to two weeks for downstream experiments. Colony-forming unit assays, including CFU-osteoblast (CFU-OB) and CFU-fibroblast (CFU-F), were performed according to established methods 38.

### Fluid shear stress (FSS) experiments

Fluid shear stress experiments were ere performed using the Streamer System STR-4000 (Flexcell International Corporation, Burlington, NC, USA) as previously described 27. MLO-Y4 cells were seeded on collagen-I-coated glass culture slips and allowed to adhere for 24-36 h prior to FSS treatment. The slips were then placed in a parallel-plate flow chamber, and cells were subjected to fluid flow at 1, 2, 5, or 10 dyne/cm² for 2 h. Static control cells were maintained under standard culture conditions in the incubator. Immediately following FSS exposure, cells were washed twice with 1×PBS, and protein and RNA samples were collected for subsequent analyses.

### ELISA assays

Serum levels of the bone formation marker P1NP were measured using the RatLaps EIA Kit (Immunodiagnostic Systems Limited, cat# AC-33F1) according to the manufacturer's protocol. Likewise, the bone resorption marker CTX-1 was quantified using the RatLaps EIA Kit (cat# AC-06F1). Briefly, serum samples were collected, diluted as required, and incubated in antibody-coated microplates. After washing to remove unbound components, enzyme-linked detection reagents were added, followed by substrate reaction and absorbance measurement using a microplate reader. Concentrations were calculated from standard curves generated with known concentrations provided in the kits.

### Bone tissue protein and RNA extraction

Mouse tibiae and femurs were carefully dissected to remove surrounding muscles. Both ends of each bone were opened, and bone marrow cells were flushed out by centrifugation at 12,000 × g. After rinsing with PBS and air-drying, bones were snap-frozen in liquid nitrogen and pulverized into a fine powder using a DEPC-treated mortar and pestle. The powdered tissue was transferred to 1.5 mL tubes, resuspended in RIPA lysis buffer (Sigma, Cat# R0278), and incubated on ice for 15 min, with gentle mixing every 5 min to ensure thorough lysis. Lysates were clarified by centrifugation at 12,000 × g for 10 min at 4 °C, and supernatants were collected for protein analysis. Protein concentrations were determined using a BCA assay. Aliquots were mixed with 6× protein loading buffer (TransGen, Cat# DL101-2), denatured at 100 °C for 10 min, and stored at -20 °C until further use.

For RNA extraction, 350 µL Buffer RLT (Qiagen RNeasy Mini Kit, Cat# 74104) was added to the bone powder, followed by an equal volume of 70% ethanol prepared with RNase-free water. The mixture was applied to RNeasy Mini spin columns and centrifuged at 12,000 g for 30-60 s. Columns were washed sequentially with Buffer RW1 and Buffer RPE (two washes), with each wash followed by centrifugation at 12,000 g. A final spin for 2 min at 12,000 g ensured complete removal of residual wash solution. RNA was eluted in 30-50 µL RNase-free water, quantified, and immediately stored at -80 °C.

### In vivo tibial loading

Mechanical loading of mouse tibiae was performed as previously described 27 using a Bose ElectroForce 3200 electroactuator (EndureTEC, Minnetonka, MN, USA). Mice were anesthetized via intraperitoneal injection of 2.5% Avertin (100 μL per 10 g body weight), and the right tibia was subjected to cyclic compressive loading at 4 Hz in a triangular waveform, with a peak force of 9.0 N for 1,200 cycles per session. The contralateral left tibia served as an internal unloaded control. Loading sessions were performed every other day over a two-week period. Micro-computed tomography (μCT) scans were acquired on Day 1 (baseline, prior to loading) and Day 15 (after the final loading session) to assess structural changes. Following the final scan, mice were euthanized, and tibiae were collected for detailed bone histomorphometric analyses, including assessment of trabecular and cortical bone parameters. This approach enables evaluation of the effects of controlled mechanical stimulation on bone remodeling *in vivo*.

### RNA extraction and qRT-PCR analysis

Total RNA was extracted from cells or tissues, reverse-transcribed into cDNA, and analyzed by quantitative real-time PCR (qRT-PCR) as previously described 39. Gene-specific primer sequences for mouse targets are listed in Table S3. Relative expression levels were calculated using standard methods, and all reactions were performed in technical triplicates to ensure reproducibility.

### siRNA and plasmid

The negative control and mouse-specific *talin1* siRNAs used in this study were synthesized by Suzhou GenePharma Co., Ltd (Suzhuo, China). Mouse negative control siRNA sequence: 5'-UUCUCCGAACGUGUCACGUTT-3'; mouse *talin1#1* siRNA sequence: 5'-GCUCCCAUCCUGUCUCCUUTT-3; mouse *talin1#2* siRNA sequence: 5'-GCUCAUUGCUGGCUACAUATT -3'.

### Western blot analysis

Cells were lysed in RIPA buffer (Sigma, USA), and 15 μg of protein per sample was separated by SDS-PAGE and transferred onto polyvinylidene fluoride (PVDF) membranes (Millipore, MA, USA). Membranes were blocked at room temperature for 30 min using QuickBlock™ Western (Beyotime) and then incubated overnight at 4 °C with primary antibodies. Following incubation with appropriate HRP-conjugated secondary antibodies (ZSGB-Bio), signals were detected using an enhanced chemiluminescence kit (ECL, Bio-Rad) and imaged with the ChemiDoc XRS system. Detailed information on the antibodies used is provided in Table S4.

### Co-immunoprecipitation (Co-IP) assay

Co-IP experiments were carried out as previously described 40. Briefly, cells were lysed in RIPA buffer (Sigma, USA) on ice for 10 min and centrifuged at 12,000 × g for 10 min at 4 °C to remove debris. The clarified lysates were incubated overnight with the specific primary antibody, followed by incubation with Protein A/G magnetic beads at room temperature for 1 h. Bead-antigen-antibody complexes were collected using a DynaMag™-2 magnet (Thermo Fisher), washed three times with IP buffer to remove non-specifically bound proteins, and resuspended in 60 μL of 1× loading buffer. Samples were then denatured at 95 °C for 5 min and analyzed by SDS-PAGE and Western blotting. This protocol allows specific isolation and detection of protein-protein interactions under native conditions.

### In vitro pull-down assays

HEK-293F cells were transiently transfected with talin1-Flag and p53-His plasmids for 60 h. Cells were lysed in 25 mM HEPES (pH 7.5), 150 mM NaCl, and protease inhibitors, and lysates were clarified by centrifugation at 14,000 × g for 30 min at 4 °C. Purified proteins were obtained via Flag and Ni-NTA affinity chromatography. For pull-down, purified talin1-Flag and p53-His were incubated at 4 °C for 2 h, followed by capture with Flag beads, extensive washing, and elution with Flag peptide. Samples were analyzed by 12.5% SDS-PAGE and Coomassie Brilliant Blue staining.

### Statistical analyses

Mice were randomly assigned to experimental groups. Sample sizes were determined based on prior experience with similar studies. IF, IHC, and histological analyses were performed and evaluated under double-blind conditions. Statistical analyses were conducted using GraphPad Prism 9. Two-way ANOVA or unpaired two-tailed Student's t-tests were applied as appropriate, with P < 0.05 considered statistically significant.

## Results

### Talin1 is critically required for FA and dendrite formation in osteocytes and down-regulated during skeletal aging in mice and humans

**Figure 1:**
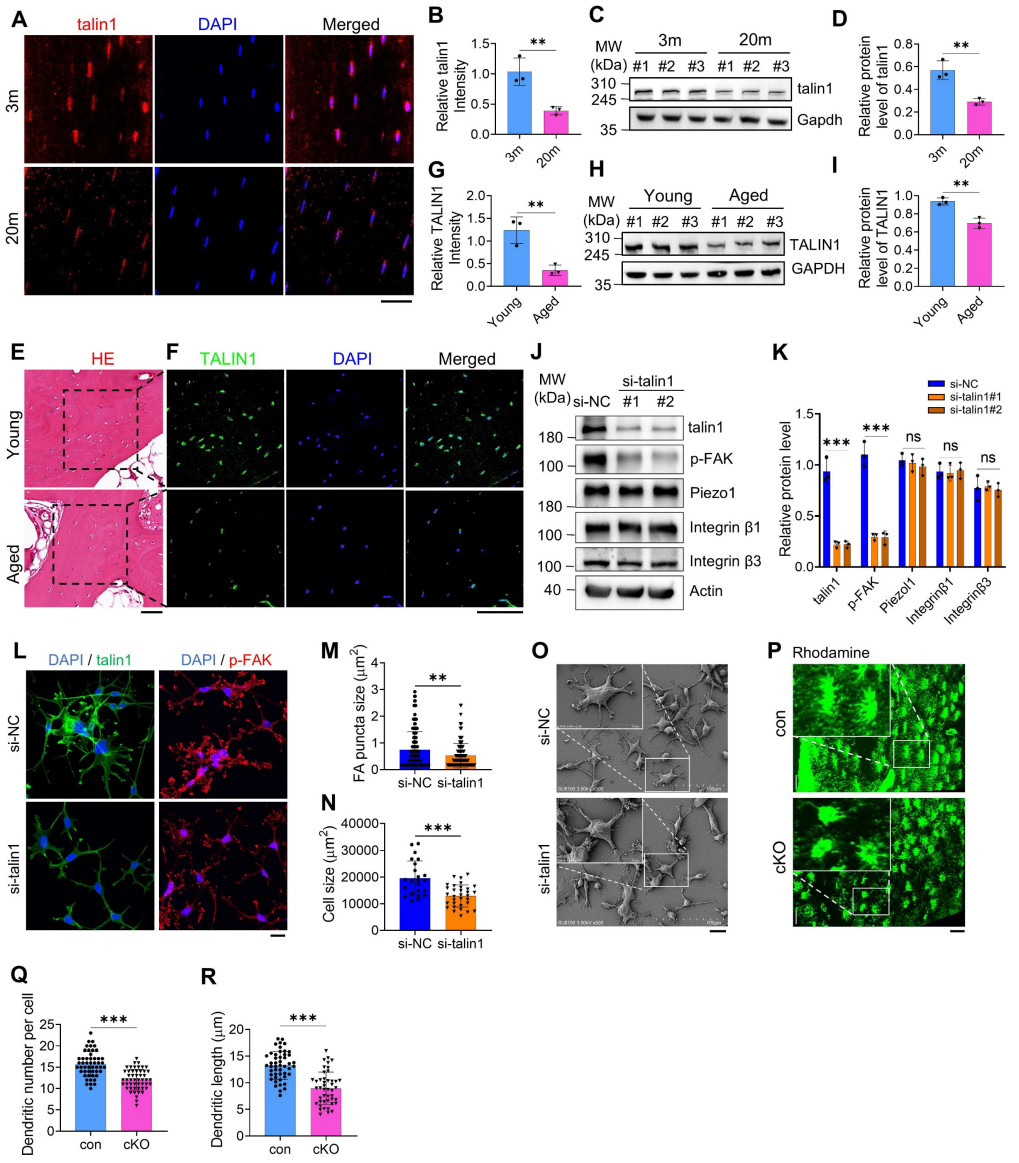
** Osteocyte talin1 regulates FA and dendrite formation and is down-regulated during skeletal aging in mice and humans. (A)** Immunofluorescence (IF) staining for talin1 expression on tibial sections fr om young (3-mo-old) and aged (20-mo-old) male mice. Scale bars, 30 μm. **(B)** Quantification data of A, N = 3, biological replicates. **(C)** Western blotting (WB). Protein extracts from cortical bone samples of young (3-mo-old male mice) and aged (20-mo-old mice) mice were subjected to western blotting for talin1 expression. **(D)** Quantification data of C, N = 3, biological replicates. **(E, F)** Hematoxylin and eosin (H/E) staining and IF staining for TALIN1 expression of young (29 years old) and aged (75 years old) human bone samples. Scale bars, 100 μm. **(G)** Quantification data of F. N = 3, biological replicates. **(H)** WB analyses for TALIN1 expression of young and aged human bone samples. **(I)** Quantification data of G, N = 3, biological replicates. **(J)** *Talin1* KD. MLO-Y4 cells were treated with si-NC (negative control) or *si-talin1#1/#2*. After 48h, cells were subjected to WB analyses for expression of the indicated proteins.** (K)** Quantification data of J. N = 3, biological replicates. **(L)** Representative IF images of si-NC- and *si-talin1*-treated MLO-Y4 cells with anti-p-FAK and anti-talin1 antibodies. Scale bars: 20 μm. **(M, N)** Quantification of L. **(O)** SEM images of si-NC and si-talin1 MLO-Y4 cells cultured on non-coated flasks. **(P)** Representative Rhodamine-stained F-actin images in tibial sections from control and cKO mice. Scale bars, 30 μm.** (Q, R)** Quantification of P. Data are presented as mean ± s.d.; n per group indicated. Statistical significance: n.s., P > 0.05; **P < 0.01; ***P < 0.001 versus controls (unpaired two-tailed Student's t test).

To explore mechanisms underlying age-related bone loss, we first analyzed RNA expression profiles from femurs of young (2-month-old) and aged (22-month-old) mice (Dataset: GSE220679). KEGG pathway enrichment identified the top 15 pathways differing between young and aged cortical bone, highlighting ECM-receptor interaction, cell adhesion, PI3K-Akt signaling, and focal adhesion (FA) pathways Figure S1A). Immunofluorescence (IF) staining of tibial sections from young (3 m) and aged (20 m) mice revealed a notable reduction of talin1 in osteocytes from aged bones (Figure 1A, B). Consistently, Western blot analyses of cortical bone (femurs and tibiae) confirmed significantly lower talin1 levels in aged versus young bones (Figure 1C, D). Examination of human trabecular bone from joint replacement surgeries, comparing young (29-35 yrs) and elderly (75-80 yrs) donors, showed a similar decline in TALIN1 expression in osteocytes within mineralized trabecular bone by IF and Western blot analyses (Figure 1E-I).

To determine whether *talin1* directly influences osteocyte function, we performed siRNA-mediated knockdown in MLO-Y4 cells, a murine osteocyte-like line. Forty-eight hours after *talin1* knockdown (KD), *talin1* expression was substantially reduced, accompanied by decreased phosphorylation of focal adhesion kinase (p-FAK) (Figure 1J, K). Importantly, total FAK levels, as well as other FA-related proteins including integrins β1 and β3 and the mechanosensitive protein Piezo1, were largely unchanged (Figure 1J, K; Figure S1B). Results from IF experiments showed reductions in FA sites and cell spreading area following *talin1* KD (Figure 1L-N). SEM images further confirmed defective cell spreading and changed morphology after *talin1* KD (Figure 1O). Functionally, cell attachment assays revealed the impaired cellular attachment and spreading on both non-coated glass surfaces and collagen-I-coated glass surfaces (Figure S2A-F).

To examine the role of talin1 in osteocyte function *in vivo*, its expression was deleted in dentin matrix protein 1 (Dmp1)-expressing cells through the breeding of floxed talin1 mice (*talin1^fl/fl^*) with 10-kb Dmp1-Cre transgenic mice (*Dmp1-Cre*). This approach generated mice lacking talin1 in osteocytes (*Dmp1-Cre; talin1^fl/fl^*), which are referred to as cKO. The results from qRT-PCR and western blotting analyses confirmed that *talin1* mRNA and protein expression was dramatically reduced in cKO cortical bone samples, which contain abundant osteocytes, but not in other soft tissues including heart, liver, spleen, lung and kidney (Figure S3A-C). Consistent with *in vitro* findings, dendrite formation in cortical osteocytes was impaired in cKO mice compared to age- and sex-matched controls (Figure 1P). Quantitative analysis over osteocyte dendrites confirmed the reduction of dendritic number and dendritic length per osteocyte of cKO mice (Figure 1Q, R).

### Talin1 ablation in osteocytes leads to osteopenia in weight-bearing bones but not in skull

**Figure 2:**
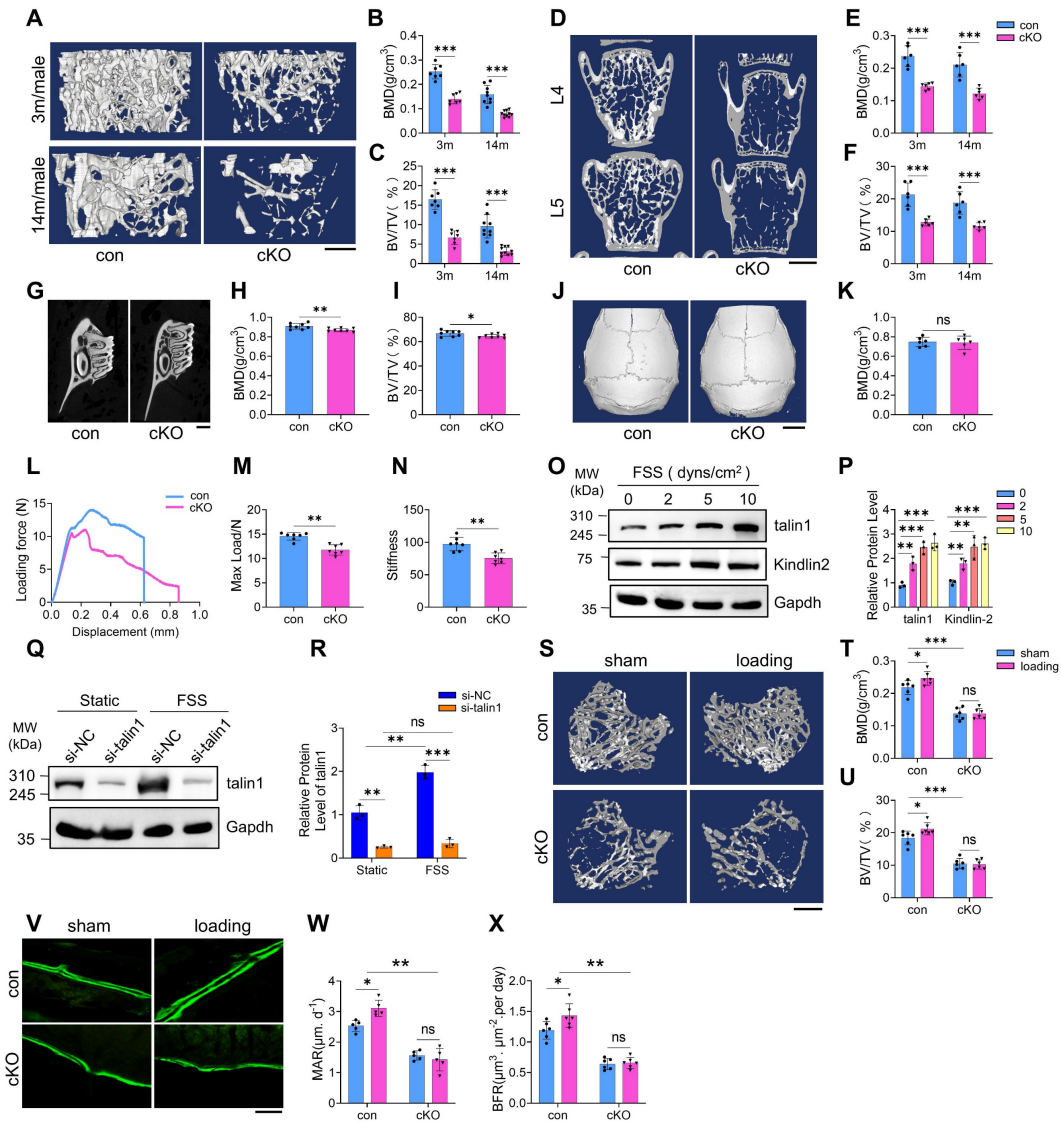
** Talin1 loss in osteocytes causes a dramatic bone loss and impairs mechanical stimulation responses in osteocytes and in bone. (A)** 3D μCT reconstruction of distal femurs from male control and cKO mice at the indicated ages. Scale bars, 500 μm.** (B, C)** Quantification of BMD and BV/TV in (A). N = 7 (3-mo males), N = 9 (14-mo control), N = 10 (14-mo cKO).** (D)** 3D μCT reconstruction of L4-L5 spines from 3-mo-old male control and cKO mice. Scale bars, 1 mm.** (E, F)** Quantitative analyses of spine BMD and BV/TV. N = 6 per group.** (G)** 3D μCT reconstruction of alveolar bone from 3-mo-old male control and cKO mice. Scale bars, 20 μm. **(H, I)** Quantification of alveolar bone parameters. N = 8 per group. **(J)** 33D μCT reconstruction of skull from male control and cKO mice. Scale bars, 2 mm.** (K)** Quantitative analysis of skull BMD and BV/TV. N = 6 per group, biological replicates. **(L)** Representative force-displacement curve for femur three point bending experiments of 3-mo-old male control and cKO mice.** (M-N)** Quantification data of maximum load and stiffness for the femurs from male control and cKO mice. N = 6 for each group, biological replicates. **(O)** WB analysis of talin1, kindlin-2 and Gapdh protein levels in MLO-Y4 cells under 0, 2, 5, and 10 dyns/cm^2^ FSS treatment. **(P)** Quantification of O. N = 3, biological replicates. **(Q)** WB analysis of talin1 expression in si-NC and *talin1* siRNA KD MLO-Y4 cells with 10 dyns/cm^2^ FSS treatment. **(R)** Quantification of Q. N = 3, biological replicates. **(S)** 3D μCT reconstruction of tibiae from 4-mo-old male control and cKO mice, with or without mechanical loading. Scale bars, 250 μm. **(T, U)** Quantitative analyses of BMD and BV/TV of in vivo loading experiments. N = 6 per group, biological replicates. **(V)** Calcein double labeling images of control and cKO loading and sham tibia sections. Scale bar, 200 μm. **(W, X)** Quantitative measurements of MAR and BFR for metaphyseal trabecular bones in the loading and sham tibial sections from control and cKO mice. N = 6 per group, biological replicates, two-way ANOVA. All results were expressed as mean ± s.d., n. s. Statistical significance: n.s., P > 0.05; **P < 0.01; ***P < 0.001 versus controls (unpaired two-tailed Student's t test).

To investigate the role of osteocyte talin1 in bone mass regulation, we conducted μCT analyses of distal femurs from 3- and 14-month-old male control and cKO mice. cKO mice displayed pronounced osteopenia at both ages, characterized by significant reductions in bone mineral density (BMD) and bone volume fraction (BV/TV), while cortical thickness (Ct.Th) remained unchanged (Figure 2A-C; Figure S3D). Similar declines in BMD and BV/TV were observed in distal femurs of 6- and 14-month-old female cKO mice (Figure S3E-G). Histological H&E staining confirmed markedly reduced trabecular bone in tibiae of cKO mice (Figure S3H). Furthermore, BMD and BV/TV were significantly decreased in the L4-L5 spine and alveolar bone of 3-month-old male cKO mice (Figure 2D-I). In contrast, non-weight-bearing skull bones were unaffected by talin1 deletion (Figure 2J, K; Figure S3I, J).

### Talin1 loss impairs the mechanical properties of bones and responses to mechanical stimuli in osteocytes and in bone

Three-point bending tests on fresh femurs from 3-month-old male mice revealed impaired mechanical properties in cKO mice, with reduced ultimate force and whole bone toughness compared to controls (Figure 2L-N). To assess whether osteocyte talin1 mediates force adaptation, we performed both in vitro and in vivo experiments. MLO-Y4 osteocyte-like cells were exposed to varying levels of fluid shear stress (FSS). Using kindlin-2 as a positive control for FSS responsiveness (27,, Western blotting showed that FSS (2-10 dynes/cm²) dose-dependently upregulated talin1 and kindlin-2 expression in MLO-Y4 cells (Figure 2O, P). Talin1 knockdown (KD) completely abolished FSS-induced talin1 upregulation (Figure 2Q, R).

*In vivo*, 4-month-old control and cKO mice underwent two weeks of cyclic loading on the right tibiae, with the contralateral left tibiae serving as unloaded controls. μCT analysis demonstrated that mechanical loading significantly increased BMD and BV/TV in control tibiae, whereas these effects were absent in cKO mice (Figure 2S-U). Consistently, calcein double labeling confirmed that loading enhanced mineral apposition rate (MAR) and bone formation rate (BFR) in control tibiae, but not in cKO tibiae (Figure 2V-X). These findings indicate that talin1 is essential for osteocyte-mediated mechanotransduction and bone adaptation to mechanical forces.

### Talin1 ablation causes abnormal bone remodeling and alters MSC differentiation fate from osteogenesis to adipogenesis in bone microenvironment

**Figure 3:**
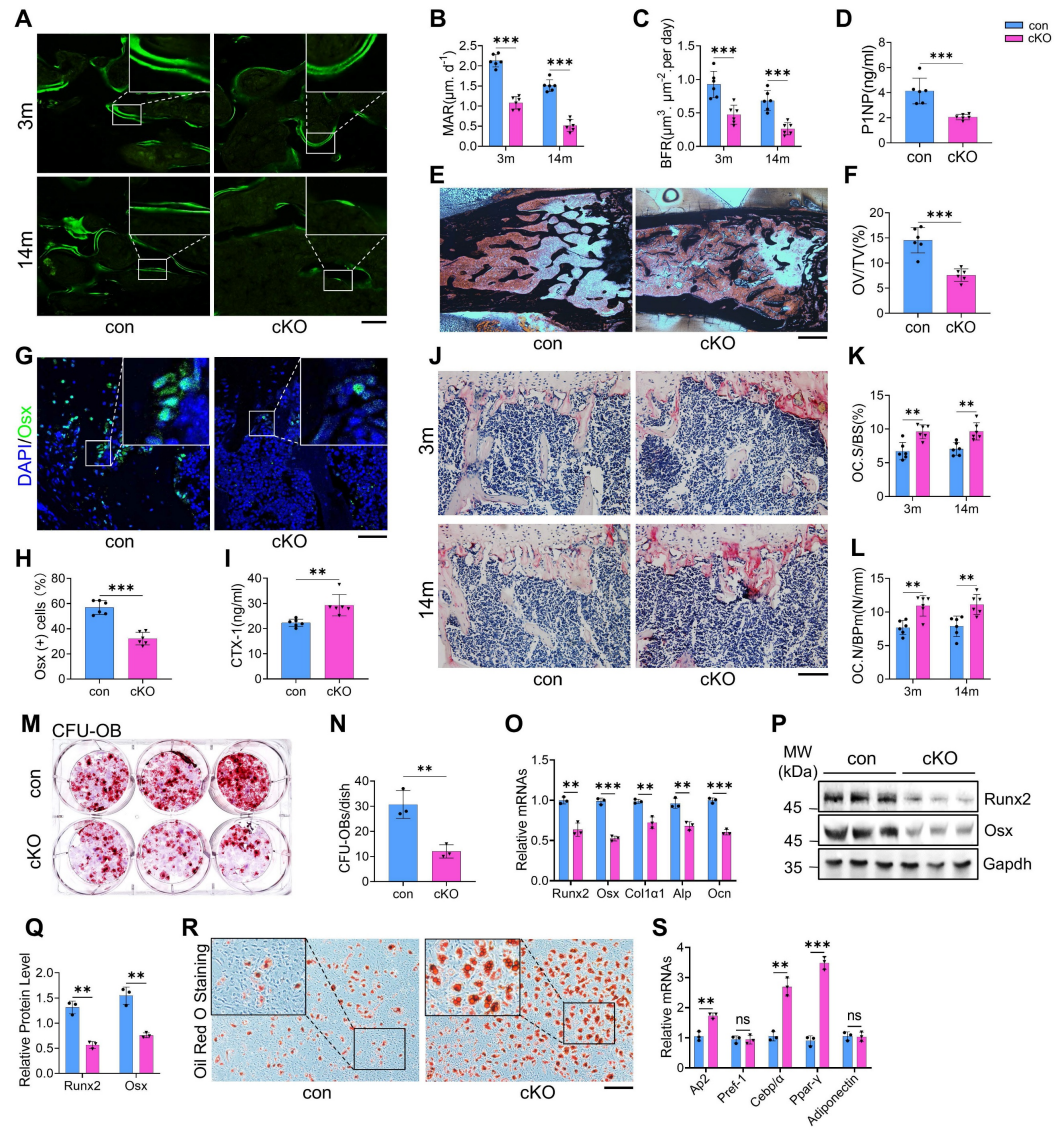
** Talin1 loss in osteocytes causes abnormal bone remodeling and switches MSC fate from osteoblastic to adipogenic differentiation in the bone microenvironment. (A)** Representative images of calcein double labeling from male control and cKO femur sections with the indicated ages. Scale bar, 200 μm. **(B, C)** Quantitative measurements of the mineral apposition rate (MAR) and bone formation rate (BFR) from calcein double labeling experiments. N = 6 per group, biological replicates.** (D)** Serum level of procollagen type 1 amino-terminal propeptide (P1NP) from 3-mo-old male control and cKO mice. N = 6 per group, biological replicates. **(E)** Representative von Kossa staining images of femoral sections from 3-mo-old male control and cKO mice. Black: Calcium in mass deposits; Red: Nuclei; Light red: Cytoplasm. Scale bar, 500 μm. **(F)** Quantification of osteoid volume/total volume (OV/TV) from von Kossa staining. N = 6 per group, biological replicates.** (G)** IF staining of osterix (Osx) in tibial sections from 3-mo-old male control and cKO mice. Scale bar, 50 μm. **(H)** Quantification of Osx-positive cells in tibiae. N = 6 per group, biological replicates. **(I)** Serum levels of collagen type I cross-linked C-telopeptide (CTX) in 3-mo-old male control and cKO mice. N = 6 per group, biological replicates.** (J)** TRAP staining of tibial sections from male control and cKO mice. Scale bar, 100 μm. **(K)** Osteoclast surface/bone surface (Oc.S/BS) and (**L**) osteoclast number/bone perimeter (Oc.N/BPm) of primary cancellous bones in the tibial sections of male control and cKO mice. Results were expressed as mean ± s.d., N = 6 per group, biological replicates. **P < 0.01 versus controls, two-way ANOVA.** (M)** Colony-forming unit-osteoblast (CFU-OB) assay from bone marrow nucleated cells of 6-mo-old male mice.** (N)** Quantitative data of M. N = 3, biological replicates.** (O)** RT-qPCR analyses of osteoblastic genes expression from 6-mo-old male control and cKO primary BMSCs after 1 week osteogenesis in vitro. N = 3 per group, biological replicates. **(P)** WB analyses of osteoblastic genes expression from 6-mo-old male control and cKO primary BMSCs after 1 week osteogenesis in vitro. N = 3 per group, biological replicates. **(Q)** Quantification of P. N = 3 per group, biological replicates. **(R)** Oil red O staining of in vitro adipogenic differentiation of primary BMSCs isolated from 6-mo-old male control and cKO mice. Scale bar, 100 μm. **(S)** RT-qPCR analyses of adipogenesis genes expression from 6-mo-old male control and cKO primary BMSCs 1 week after adipogenesis induction in vitro. N = 3 per group, biological replicates. All results were expressed as mean ± s.d., n. s. Statistical significance: n.s., P > 0.05; **P < 0.01; ***P < 0.001 versus controls (unpaired two-tailed Student's t test).

To explore the mechanisms underlying bone loss in cKO mice, we evaluated osteoblast and osteoclast formation and function. Talin1 deletion markedly impaired bone formation, as evidenced by significant reductions in mineral apposition rate (MAR) and bone formation rate (BFR) in the femoral metaphyseal cancellous bones of both 3- and 14-month-old male cKO mice (Figure 3A-C). Serum levels of procollagen type 1 amino-terminal propeptide (P1NP), an in vivo marker of bone formation, were also decreased in cKO mice (Figure 3D). Von Kossa staining revealed a notable reduction in osteoid volume/tissue volume (OV/TV) in cKO bones, indicating defective mineralization (Figure 3E, F). Immunofluorescence staining for osteoblast markers, including osterix (Osx) and osteocalcin (Ocn), showed fewer osteoblasts and progenitors on bone surfaces in cKO mice compared to controls (Figure 3G, H; Figure S4A, B).

Conversely, osteoclast formation and resorptive activity were elevated in cKO mice. Serum collagen type I cross-linked C-telopeptide (CTX) levels were significantly higher than controls (Figure 3I). TRAP staining revealed increases in osteoclast surface/bone surface (Oc.S/BS) and osteoclast number/bone perimeter (Oc.Nb/BPm) in primary cancellous bones of cKO mice (Figure 3J-L). In vitro assays using primary bone marrow monocytes (BMMs) confirmed enhanced osteoclastogenesis in cKO cultures, with ~50% more TRAP-positive multinucleated cells (≥3 nuclei per cell) than controls Figure S4C, D).

Bone marrow colony-forming assays showed that talin1 loss reduced colony-forming unit-osteoblasts (CFU-OB) without affecting colony-forming unit-fibroblasts (CFU-F) (Figure 3M, N; Figure S4E, F). In vitro differentiation of primary bone marrow stromal cells (BMSCs) further demonstrated impaired osteoblast differentiation in cKO mice, as indicated by reduced ALP activity (Figure S4G, H) and downregulation of osteoblast-specific genes (Figure 3O-Q). In contrast, adipogenic differentiation was enhanced in cKO BMSCs, with increased oil red O staining and elevated expression of adipogenic markers, including Ap2, Cebp, and Ppar-γ (Figure 3R, S). Collectively, these findings indicate that talin1 deficiency in osteocytes disrupts bone remodeling by suppressing bone formation while promoting bone resorption and adipogenesis.

### Talin1 loss greatly accelerates cellular senescence in MLO-Y4 osteocyte-like cell line, primary osteocytes, and osteocytes in bone

Osteocyte senescence is considered a key contributor to age-related bone loss (41, 42. To evaluate this, we examined p53, a well-established marker of cellular senescence, in human trabecular bone samples from young (29-35 years) and elderly (75-80 years) donors. Immunofluorescence and Western blot analyses revealed a marked increase in p53 expression in osteocytes within the mineralized trabecular bone of aged individuals compared to young donors Figure S5A-C).

**Figure 4:**
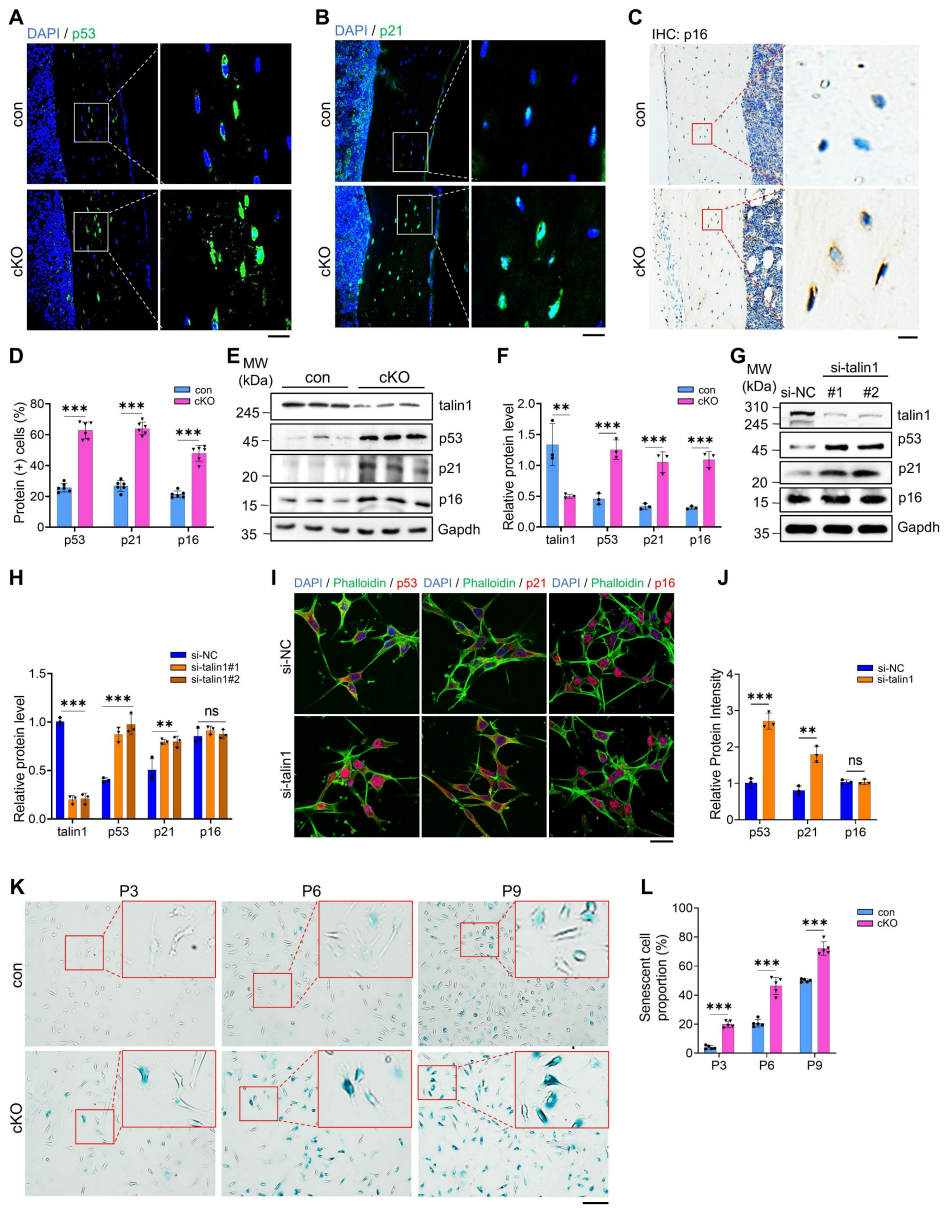
** Talin1 loss greatly accelerates cellular senescence in MLO-Y4 osteocyte-like cell line, primary osteocytes, and osteocytes in bone. (A-C)** IF and immunohistochemical (IHC) staining for p53, p21 and p16 expression on 6-mo-old male control and cKO tibial sections. Scale bar, 50 μm. **(D)** Quantification of A-C. N = 6 per group, biological replicates. **(E)** Western blotting for talin1, p53, p21 and p16 proteins extracted from cortical bones of 3-mo-old male control and cKO mice. **(F)** Quantification of E. N* =* 3 per group.** (G)** Western blotting analyses of talin1, p53, p21 and p16 proteins in MLO-Y4 cells transfected with control or *si-talin1* siRNA. **(H)** Quantification of G. N* =* 3 per group, biological replicates. **(I)** IF staining for p53, p21 and p16 expression in MLO-Y4 cells with and without *talin1* KD. Scale bar, 50 μm. **(J)** Quantification of I. N* =* 3 per group, biological replicates. **(K)** Representative images of SA-β-Gal staining with primary osteocytes derived from control and cKO mice. Scale bar, 50 μm. **(L)** Quantification of K. N* =* 3 per group, biological replicates. All results were expressed as mean ± s.d., n. s. Statistical significance: n.s., P > 0.05; **P < 0.01; ***P < 0.001 versus controls (unpaired two-tailed Student's t test).

To determine whether talin1 loss promotes osteocyte senescence in bone, we performed IHC and IF staining of bone sections of the two genotypes and found that the numbers of osteocytes expressing cellular senescence markers, including p53, p21 and p16, were all dramatically increased in cKO relative to those in control bones (Figure 4A-D). Results from western blotting confirmed increases in expression of p53, p21 and p16 proteins in cKO versus control cortical bones (Figure 4E, F). Results from both western blotting and IF staining revealed that *talin1* KD up-regulated p53 and p21, but not p16, in MLO-Y4 cells (Figure 4G-J). We further isolated primary osteocytes from cortical bones of 6-month-old control and cKO mice using a sequential digestion method (43. We performed the β-galactosidase staining of different passages of primary osteocyte cultures from both control and cKO mice and found that talin1 loss greatly increased the percentages of osteocytes expressing high level of β-galactosidase (Figure 3K, L).

### Talin1 directly binds to and retains p53 in cytoplasm for ubiquitin-proteasomal degradation and talin1 loss greatly elevates the level of nuclear p53

**Figure 5:**
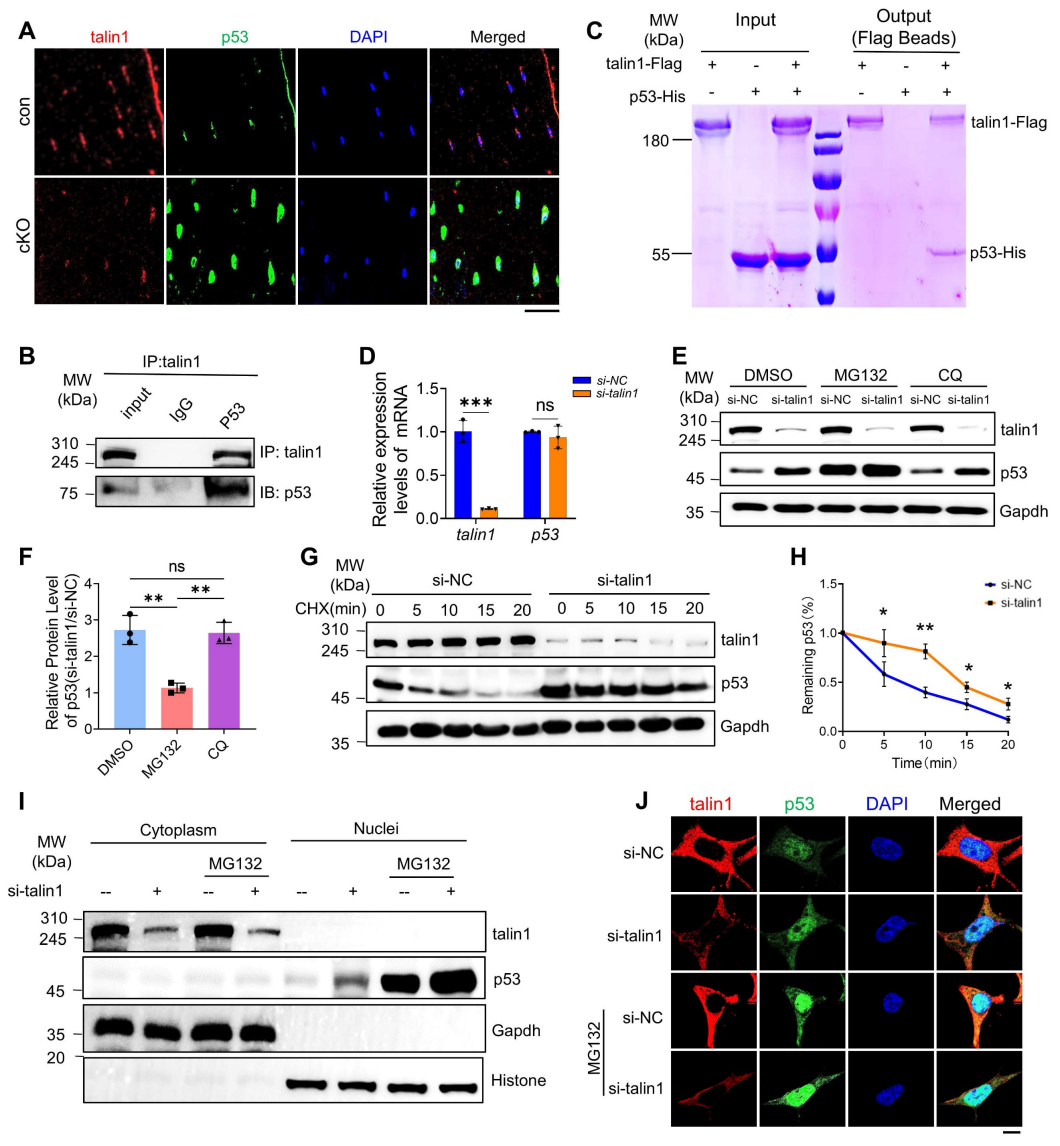
** Talin1 directly interacts with p53 and retains p53 in cytoplasm to promote its ubiquitin-proteasomal degradation. (A)** IF staining for talin1 and p53 expression on 6-mo-old male control and cKO mice tibial sections. Scale bar, 30 μm.** (B)** In vivo co-immunoprecipitation (co-IP) assay. **(C)** In vitro pull-down assay of talin1-Flag and p53-His proteins. **(D)** RT-qPCR analyses of *talin1* and *p53* mRNA levels with and without *si-talin1* KD in MLO-Y4 cells. N* =* 3 per group, biological replicates. **(E)** WB analysis of talin1 and p53 expression with and without *si-talin1* KD under the treatment of DMSO, MG132 or CQ. **(F)** Quantification of E. N* =* 3 per group, biological replicates. **(G)** WB analysis of CHX treated MLO-Y4 cells with si-NC and *si-talin1* KD at indicated time. (**H**) Quantification of the remaining p53 protein level in G. **(I)** WB analysis of talin1 and p53 in cytoplasmic and nuclear fractions with or without *si-talin1* KD and MG132 treatment. Gapdh and histone were used as markers for the cytoplasmic and nuclear proteins, respectively.** (J)** IF staining for talin1 and p53 in MLO-Y4 cells with or without *si-talin1* KD and MG132 treatment. Scale bar, 5 μm. All results were expressed as mean ± s.d., n. s. Statistical significance: n.s., P > 0.05; **P < 0.01; ***P < 0.001 versus controls (unpaired two-tailed Student's t test).

Because the p53 transcription factor is known to play a critical role in the regulation of cellular senescence, we next focus our studies on determining how talin1 loss up-regulates p53 in osteocytes. Results from IF staining showed that both talin1 and p53 proteins were partially co-expressed in the cytoplasm of osteocytes from cortical bones (Figure 5A). To determine if talin1 interacts with p53, we performed immunoprecipitation assays using protein extracts from MLO-Y4 cells with an anti-talin1 antibody and detected a clear enhancement of p53 protein in the immunoprecipitates (Figure 5B). To determine whether talin1 can directly interact with p53, we purified talin1-Flag and p53-His proteins from HEK-293F cells overexpressing both proteins. Results from the pull-down assay revealed that talin1-Flag could directly interact with p53-His (Figure 5C). Moreover, we found that *talin1* KD did not affect the level of* p53* mRNA (Figure 5D), suggesting that a post-transcriptional mechanism is involved in this regulation. To determine if this was the case, we tried to decipher whether the ubiquitin-proteasome pathway or autophagosome protein degradation participates in this talin1-p53 regulation in osteocytes. We utilized proteasome inhibitor MG132 and autophagy inhibitor chloroquine (CQ) to treat MLO-Y4 cells with and without *talin1* KD. Results showed that MG132, but not CQ, dramatically increased the basal level of p53 protein in MLO-Y4 cells (Figure 5E, F). Furthermore, MG132, but not CQ, essentially abolished the *talin1* KD-induced increase of p53 protein (Figure 5E, F). Collectively, these results suggest that it is the ubiquitin-proteasome pathway rather than the autophagosome protein degradation pathway that controls both basal and talin1-mediated regulation of p53 protein levels in these cells. We further conducted cycloheximide (CHX) chase assay to visualize p53 protein degradation kinetics over different timepoints in MLO-Y4 cells with or without *talin1* KD. Results showed that *talin1* KD up-regulated the expression of p53 protein and reduced its degradation in MLO-Y4 cells (Figure 5G, H). These data together suggest that talin1 enhances p53 ubiquitination and degradation. Since p53, as a transcription factor, exerts its function mainly in the nuclei, we next determined its protein levels in the cytosol and nuclear extracts from MLO-Y4 cells with and without *talin1* KD in the presence and absence of MG132 treatment. Results showed that the level of p53 was extremely low in the cytoplasm in MLO-Y4 cells with or without MG132 treatment. *Talin1* KD markedly elevated the level of nuclear p53, which was further increased by MG132 treatment (Figure 5I). As expected, talin1 was detected in cytosol but not in nuclei. IF staining of cultured MLO-Y4 cells confirmed that *talin1* KD increased the expression of p53 in nuclei, which was further increased by MG132 treatment (Figure 5J). Collectively, our results support the notion that talin1 down-regulates p53 by directly binding to and retaining p53 in the cytoplasm for subsequent ubiquitin-proteasomal degradation.

### Genetically deleting p53 expression in osteocytes restores the low bone mass phenotype, impaired bone-forming activity, and enhanced cellular senescence caused by talin1 loss in mice

**Figure 6:**
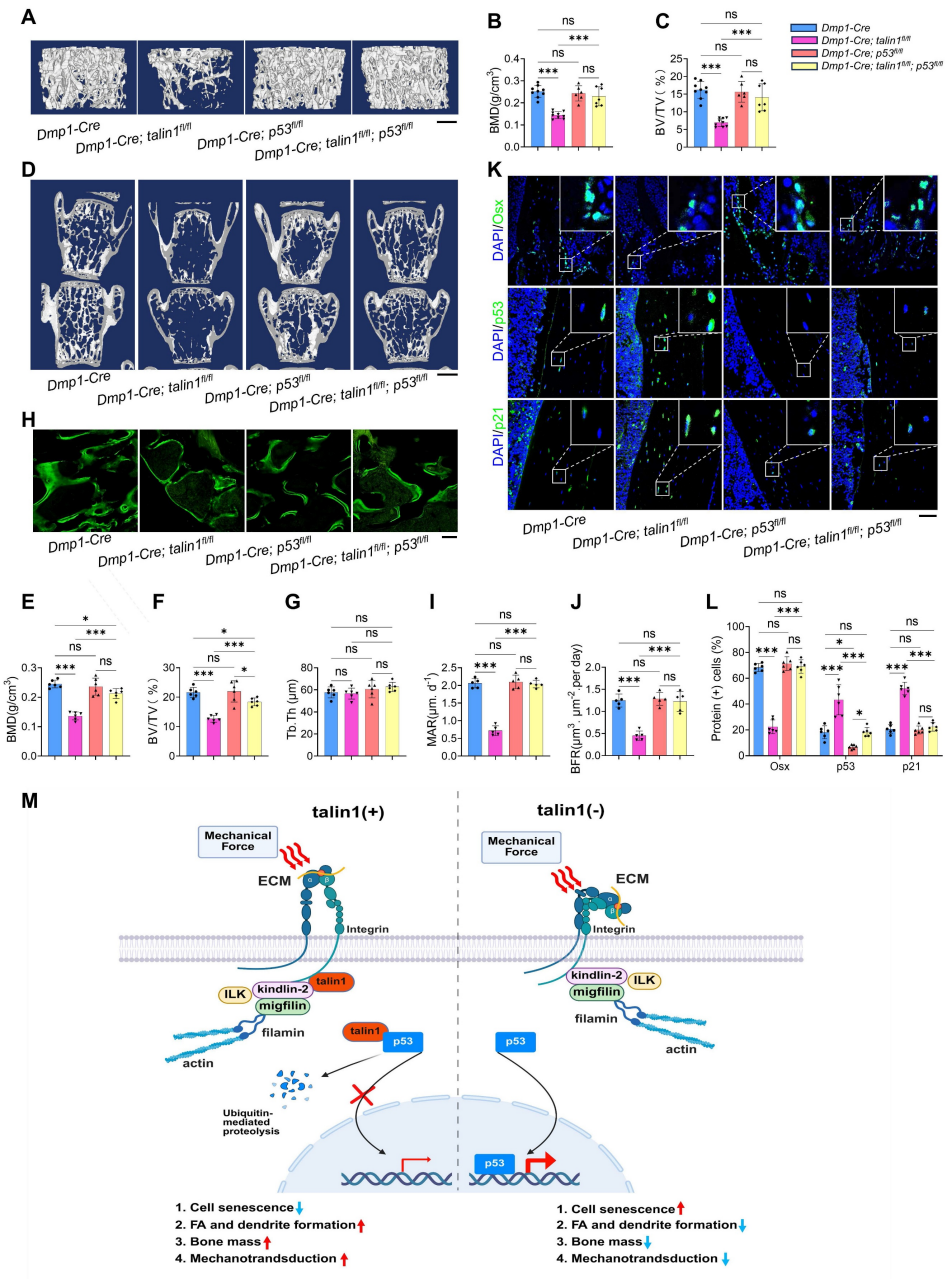
** Deleting p53 in osteocytes restores the low bone mass phenotype, impaired bone-forming activity, and enhanced cellular senescence caused by talin1 loss in mice. (A)** 3D μCT reconstruction of tibiae from 3-mo-old male mice of indicated genotypes. Scale bar, 500 μm. **(B, C)** Quantification of tibial BMD and BV/TV. N = 6-9 per group; ordinary one-way ANOVA.** (D)** 3D μCT reconstruction of L4-L5 spines from 3-mo-old male mice. Scale bar, 1 mm. **(E-G)** Quantitative analyses of spinal BMD, BV/TV, and thickness. N = 6 per group; ordinary one-way ANOVA. **(H)** Representative images of femur sections of calcein double labeling in four groups. Scale bars, 200 μm. **(I)** Quantitative measurements of mineral apposition rate (MAR) and **(J)** bone formation rate (BFR) of double calcein labeling experiments. **(K)** IF staining for osterix (Osx), p53 and p21 expression of the tibial sections from3-mo-old male mice with indicated genotype. Scale bar, 50 μm. **(L)** Quantification analysis of osterix-positive, p53 positive and p21 positive cells in tibial sections from 3-mo-old male mice with indicated genotype. N = 6 per group, biological replicates. All results were expressed as mean ± s.d., n. s. *P* > 0.05, *P < 0.05, **P < 0.01, ***P < 0.001 versus controls, ordinary one-way ANOVA.** (M)** Working model.

We next tested our hypothesis that talin1 loss reduces bone mass by up-regulating p53 in osteocytes. To this end, we determined the effects of p53 deletion in osteocytes on the osteopenic phenotype in talin1 cKO mice. We generated the *Dmp1-Cre; talin1^fl/fl^; p53^fl/fl^* mice in which both talin1 and p53 expression was deleted in osteocytes and other genotypes, including *Dmp1-Cre* (as control), *Dmp1-Cre; talin1^fl/fl^*, and *Dmp1-Cre; p53^fl/fl^* mice. Results from μCT analyses of the femur samples of 3-mo-old mice of the four groups showed that mice with single *p53* loss in osteocytes had the comparable BMD and BV/TV as control mice (Figure 6A-C). Importantly, *p53* deletion largely reversed the osteopenia of *talin1* cKO mice in the double knock out mice (Figure 6A-C). Histological analysis using H&E staining of tibial sections confirmed that p53 ablation reversed the low bone mass in talin1-deficient mice Figure S6A). Consistently, *p53* inactivation also reversed the osteopenic phenotype in the spine (L4-L5) of talin1 cKO mice (Figure 6D-G).

Next, we wondered whether the bone-forming capacity could be revised by *p53* deletion in *talin1* cKO mice with double calcein labeling experiments. As expected, p53 inactivation led to significant increases in the MAR and BFR in double knockout mice (Figure 6H-J), suggesting that *p53* deletion restored the impairment in bone formation in *talin1* cKO mice. Since loss of talin1 in osteocytes results in morphological defects, we next investigated whether deletion of p53 could rescue these abnormalities in conditional knockout (cKO) mice. To this end, we performed double staining of tibial cortical bone sections from 3-month-old control mice, two single knockout models, and double knockout mice, using DAPI to label nuclei and Rhodamine to visualize the actin cytoskeleton.

As shown in Figure S 6B, osteocytes from p53 single knockout mice exhibited cellular morphology comparable to that of control mice, whereas osteocytes from double knockout mice displayed reduced dendrite number and length, similar to those observed in talin1 single knockout osteocytes. Quantitative analyses further confirmed that loss of p53 neither promoted dendritic formation nor rescued the morphological defects caused by talin1 deletion (Figure S6C, D). Interestingly, however, p53 deficiency in osteocytes reduced the elevated osteocyte number observed in cortical bone sections of talin1-deficient mice (Figure S6E). Moreover, we applied IF staining against osterix (Osx) for osteoblast cells on the tibial cortical bone sections from 3-month-old control mice, two single knockout mice and double knockout mice. As presented in Figure. 6K, osteocytes with single p53 deletion displayed similar Osx-positive cells as control mice, and the double knockout osteocytes displayed comparable numbers of Osx-positive cells as control mice. Quantitative data further confirmed that loss of p53 in osteocytes rescued the impaired bone formation caused by *talin1* deletion (Figure 6K). In addition, we also detected the p53 and p21 protein expression with IF experiments on the tibial sections from four groups of genotypes. IF images and quantitative analysis further showed that *p53* deletion in osteocytes reduced the elevated markers of cell senescence genes caused by *talin1* knock-out in osteocytes (Figure 6K-L).

## Discussion

FAs serve as critical signaling hubs that regulate mechanotransduction and cellular responses to external forces, playing a vital role in maintaining bone homeostasis. Talin1, a key FA adaptor protein, has been extensively studied in tumor cell proliferation, migration, metastasis and relapse (44, 45. It has been recognized as a potential biomarker for multiple types of cancers, including melanoma skin cancer 8 and high-grade ovarian cancer 46. Beside cancer cells, talin1 has been widely accepted as an important mechanotransductive proteins in different types of cells in diverse tissues, such as podocytes in the kidney 19 and cardiomyocytes in the muscle 20. Considering the ubiquitous expression of talin1 in various tissues, its physiological functions in mammals are incompletely understood. In bone environment, talin1 has been reported with crucial functions in osteoclast differentiation 47 and osteoclast-mediated bone resorption 48. In this study, we reveal a previously unknown role of talin1, via its expression in osteocytes, in modulation of bone mass and mechanotransduction.

Our findings in this study demonstrate that deletion of talin1 in osteocytes leads to a dramatic bone loss and impairs bone mechanical properties, suggesting its crucial functions in maintaining skeletal homeostasis. Besides the abnormal osteocyte phenotype in cKO mice, the bone microenvironment was largely altered including reduced bone-formation of osteoblasts, enhanced bone-resorption of osteoclasts and preferred adipogenesis over osteogenesis of BMSCs (Figure 3. M-S). All of these changes in cKO bone may contribute to diverse paracrine factors released by all cell types mentioned above which together generate a complex and dynamic bone microenvironment. The difference between in vivo bone microenvironment and in vitro cell culture system may explain the different activation of p16 in vivo and in vitro results.

Given that osteocytes are the primary mechanosensors in bone, the disruption of FA-mediated mechanotransduction due to talin1 depletion may compromise the ability of bone cells to respond to mechanical stimuli, ultimately leading to bone loss. This aligns with previous studies highlighting the roles of several FA-related proteins, including integrin β1, kindlin-2 and pinch1/2, in control of bone mass, reinforcing the importance of FA integrity in regulation of the skeletal formation and homeostasis 27-30, 49, 50. Published data demonstrate that talin1 activation is tightly associated with integrin and kindlin proteins through FA signaling 51. In current study, we showed that talin1 is indispensable in osteocytes for the mechanotransduction of force-induced bone formation. Published data from our and other groups suggest that the potential upstream signaling behind talin1 activation and function during mechanotransduction and aging could be FA pathway. The external force could stimulate integrins β1 and β3 on the osteocyte membrane, which further leads to a conformational change of integrin subunits and actively recruits talin1, kindlin-2 and other responsive proteins for sequential signaling cascades. In current study, we extended this idea further and indicated that the downstream of talin1 signal is p53 in osteocytes. Our in vitro FSS data showed that talin1 was up-regulated by FSS stimulation, which may further down-regulate p53 in osteocytes. Once the talin1 expression was knocked down, its suppression of p53 upon FSS was released. Together, these data suggest that mechanical force contributes to osteocyte survival by suppressing p53 expression through talin1.

Since FA is a large macromolecular complex formed by more than 100 proteins, talin-p53 interaction may involve in other protein players. During cell migration, the FA plaques undergo a dynamic transition from nascent adhesion into mature adhesion, and finally disassembly process. The FA components included structural proteins (integrin, talin, vinculin etc.), signaling proteins (FAK, Src), adaptor (paxillin) and cytoskeletal components (G-actin, F-actin) also undergo robust interaction, assembly and disassembly processes 52. Published data demonstrate that FAK recruits talin through a direct binding interaction at nascent adhesions 53, which is independently of direct talin binding to β1 integrin 54. Moreover, talin localizes to more mature adhesions later on, even in the absence of FAK 55. These data suggest that FAK is upstream of talin during nascent adhesion formation, but independent of talin signaling at mature adhesion stage 56. Recent studies have demonstrated that disrupting the interactions between talin and either vinculin or FAK impairs YAP nuclear translocation and transcriptional activity in fibroblasts and human mesenchymal stem cells (hMSCs) 57. However, vinculin-talin binding, but not FAK-talin binding, is essential for nuclear size control in these cells 57. In the current study, we notice that transient knockdown of talin1 in osteocytes results in reduced FAK and p-FAK expression, which may explain the reduced FA site, cell spreading and attachment in siTalin1 cells.

In addition to its structural role in FA assembly, talin1 has emerged as a key regulator of cellular signaling pathways, including those governing cell survival and senescence. In bone, osteocyte morphology and viability controlled by cell apoptosis and senescence are tightly linked with its bone remodeling ability 58. p53, a well-established regulator of cellular senescence, has been reported with anti-osteogenic functions in bone mesenchymal progenitor cells by suppressing OPG 59. Furthermore, Wnt/ß-catenin-mediated p53 suppression was reported to be essential for osteogenesis of mesenchymal progenitor cells 60. Here, in this study, we identify a direct interaction between talin1 and p53 that controls p53 degradation and nuclear translocation. Protein p53 has been referred to as the “Guardian of the Genome” considering its essential roles in response to stresses that can disrupt the fidelity of DNA replication and cell division 61. This important transcription factor regulates the cellular program of cell cycle arrest, cellular senescence and apoptosis in various tissue backgrounds. Since osteocyte apoptosis is tightly linked to aging and sex steroid deficiency-related bone loss 62, p53 is widely recognized as a pro-apoptotic biomaker in aging- 63 and estrogen-deficiency-related 64 osteocyte apoptosis and bone loss. It would be interesting to further investigate the potential downstream players of talin1-p53 axis in osteocyte senescence (including CDK4/6, Cyclin D) and/or osteocyte apoptosis (including Bcl2, BAX, Puma) in further research.

Previous reports showed the protein-protein interaction between the N-terminal fragment of FAK and p53 during cancer cell tumorigenesis 65. Moreover, p53 also binds to the FAK promoter to inhibit FAK transcription in vitro 66-68 and in vivo 69. Animal studies further showed that FAK inactivation results in p53- and p21-dependent mesodermal cell growth arrest during mouse development 70. This binding between FAK and p53 facilitates p53 ubiquitination in both nuclear region and potential cytoplasmic region in cancer cells 70. In current study, we find that talin1 loss leads to increased p53 expression in osteocytes, accelerating cellular senescence and impairing bone formation. This suggests that talin1 exerts a protective effect against osteocyte senescence. The fact that genetic deletion of p53 in osteocytes rescued the osteopenic phenotype caused by talin1 loss further supports the functional link between FA disruption, p53 activation, and bone deterioration. Our data showed that in osteocytes, talin1 directly interacts with p53 protein in the cytoplasm and enhances p53 degradation in the cytoplasm, which prevents its nuclear translocation and downstream signaling. Moreover, our in vitro data showed that transient knockdown of talin1 through siRNA in MLOY4 cells caused no changes in total FAK expression, but a reductions of p-FAK in these cells. It would be interesting to further decipher the tissue specific regulation in talin, FAK and p53 interaction.

In this study, we identify talin1 as a potential suppressor of age-related osteoporosis and a mediator of force-induced bone remodeling by inhibiting p53-mediated cellular senescence, although the downstream effectors of p53 require further investigation. Cellular senescence is primarily regulated by the p53/p21 and p16/Rb tumor suppressor pathways 71. Specifically, p21 mediates p53-induced cell cycle arrest at the G1/S or G2/M checkpoints by interacting with various apoptosis-related proteins, including caspases, thereby promoting senescence through apoptosis inhibition 72. In contrast, p16 and the retinoblastoma (Rb) protein family play a central role in senescence maintenance 72. Thus, senescence can be prevented by p53 inactivation prior to p16 upregulation, but once p16 is highly expressed, p53 downregulation cannot reverse cell cycle arrest. Consistent with this, cortical bone from cKO mice exhibited elevated levels of p53, p21, and p16 compared to controls. In MLO-Y4 cells, siRNA-mediated talin1 knockdown increased p53 and p21 expression without affecting p16. These findings suggest that both senescence initiation and maintenance are enhanced in cKO mice, whereas transient siTalin1 treatment primarily influences the initiation phase of cellular senescence. Further studies are needed to delineate the downstream signaling of the talin1-p53 axis.

The identification of talin1 as a critical regulator of bone remodeling and osteocyte senescence provides a promising avenue for therapeutic intervention in microgravity-induced bone loss, disused osteoporosis, and age-related bone loss. Given that p53 up-regulation contributes to bone deterioration caused by the absence of talin1 in osteocytes, targeting the talin1-p53 axis may offer new strategies to mitigate osteocyte senescence and bone loss. Small molecules or peptides that stabilize talin1 integrity or modulate p53 expression level and/or nuclear translocation could serve as potential therapeutic candidates for osteoporosis treatment. Moreover, future studies should explore whether enhancing talin1 expression or function in osteocytes can prevent or reverse age-associated bone degeneration.

Based on findings from this and other studies, we propose a working model in which talin1 functions as a mechanotransduction mediator as well as a cell senescence suppressor in osteocytes (Figure 6M). In healthy young and adult skeleton, talin1 is highly expressed in osteocytes where it facilitates FA formation and stability and promotes integrin signaling, which contributes to osteocyte mechanosensing and bone remodeling. In addition, talin1 directly and potently binds to p53 protein in cytoplasm, which prevents p53 nuclear translocation of and facilitates its ubiquitination and degradation in cytoplasm. Together, talin1 inhibits osteocyte senescence and maintains normal bone remodeling. In contrast, in aged or unloaded skeleton, talin1 expression in osteocytes is largely down-regulated, which impairs FA assembly and mechanosensing and causes p53 up-regulation and accumulation in nuclei, ultimately resulting in osteocyte senescence, abnormal bone remodeling and bone loss, and compromised mechanotransduction in bone. This model integrates FA dynamics, osteocyte senescence, and bone remodeling into a unified framework, offering a novel perspective on age- and microgravity-related bone loss.

Taken together, our study establishes talin1 as a crucial regulator of osteocyte function, linking FA integrity with p53-mediated cellular senescence in bone remodeling. The findings provide valuable insights into the molecular mechanisms of age- and unloading-related bone loss and lay the foundation for potential therapeutic strategies targeting the talin1-p53 axis to improve bone health in aging populations.

## Supplementary Material

Supplementary figures and tables.
