# Supplementary material for: The talin1-p53 axis inhibits osteocyte senescence to promote bone mass and mediate skeletal adaptation to mechanical stimulation
Source: Theranostics. 2026 Jan 1;16(1):465–82. doi: 10.7150/thno.123532 (PMC12665141; doi:10.7150/thno.123532)
Supplement: Supplementary file 1 — Supplementary figures and tables. [file thnov16p0465s1.pdf]

Supplementary data

Table S1: Medical Record Table.

Patient	Sex	Age	Past Medical History	Present Illness
Young#1	Male	29	None	Fracture caused by a traffic accident leading to extensive damage of the articular surface
Young#2	Female	33	Mild fatty liver	Femoral head necrosis caused by long-term excessive alcohol consumption
Young#3	Male	35	None	Irreversible joint destruction caused by rheumatoid arthritis
Aged#1	Male	75	Hypertension for over 5 years, regularly taking antihypertensive medication	Left femoral neck fracture due to hip pain after a fall
Aged#2	Male	79	History of hypertension for more than 10 years, self-medicated	Right hip pain and restricted mobility after a fall
Aged#3	Female	80	Bradycardia for 5 years treated with a pacemaker; history of diabetes for 5 years, untreated systematically	Right hip trauma caused by a fall

Table S2: Primers used for genotyping analysis.

Gene name	Forward	Reverse
Dmp1-Cre	GATCTCCGGTATTGAAACTCCAGC	GCTAAACATGCTTCATCGTCGG
talin1	AGTGGGTGTTGTTGCTCTTCATAA	CTCCACTTTCCCCACTCGGTTGT
p53	GAGCATGGAAGTAAGACCCCTTCT	GACAGGGTTTCTCTATGTAGCCCT

Table S3: primers used for qRT-PCR analysis.

Gene name	5' primer	3' primer
Runx2	AACGATCTGAGATTTGTGGGC	CCTGCGTGGGATTTCTTGGTT
Osx	ATGGC GTCCTCTCTGCTTG	TGAAAGGTCAGCGTATGGCTT
Col1α1	GCTCCTCTTAGGGGCCACT	CCACGTCTCACCATTGGGG
Alp	CCAACTCTTTTGTGCCAGAGA	GGCTACATTGGTGTTGAGCTTTT
Ocn	AGGGAGGATCAAGTCCCG	GAACAGACTCCGGCGCTA
Ap2	GGGGCCAGGCTTCTATTCC	GGAGCTGGGTTAGGTATGGG
Pref-1	CCCAGGTGAGCTTCGAGTG	GGAGAGGGGTACTCTTGTTGAG
Cebp/α	CAAGAACAGCAACGAGTACCG	GTCACTGGTCAACTCCAGCAC
Ppar-γ	TCGCTGATGCACTGCCTATG	GAGAGGTCCACAGAGCTGATT
Adiponectin	TGTTCCTCTTAATCCTGCCCA	CCAACCTGCACAAGTTCCCTT
talin1	GGCCCTCCCAACGACTTT	AGCCTCTAGCCAGATGCCTTT
Gapdh	AGGTCGGTGTGAACGGATTTG	TGTAGACCATGTAGTTGAGGTCA
p53	CTCTCCCCCGCAAAAGAAAAA	CGGAACATCTCGAAGCGTTTA

Table S4: antibody information.

Antibody	Company	Catalog #	Application/Dilution
talin-1	Proteintech 14168-1-AP WB	14168-1-AP	WB (1:1000) IF (1:200)
Gapdh	ZSGB-BIO	TA-08	WB (1:1000)
p-FAK	Abcam	ab81298	WB (1:1000) IF (1:100)
Piezo1	Proteintech	15939-1-AP	WB (1:500)
Integrin β1	Abcam	ab95623	WB (1:1000)
Integrin β3	Cell Signaling Technology	13166	WB (1:1000)
Actin	TransGene	HC201	WB (1:2000)
Kindlin2	Millipore	MAB2167	WB (1:1000)
Runx2	Cell Signaling Technology	12556	WB (1:1000)
Osx	Abcam	ab22552	WB (1:1000) IF (1:300)
Ocn	Proteintech	23418-1-AP	IF (1:200)
p53	CST	2524S	WB (1:1000) IF (1:200)
p21	Abcam	ab188224	WB (1:1000) IF (1:200)
P16	Abcam	ab211542	WB (1:1000) IHC/IF (1:200)
Histone	Proteintech	68345-1-Ig	WB (1:1000)
phalloidin-488	Invitrogen™	A12379	IF (1:500)

Supplementary Figures and Figure Legends

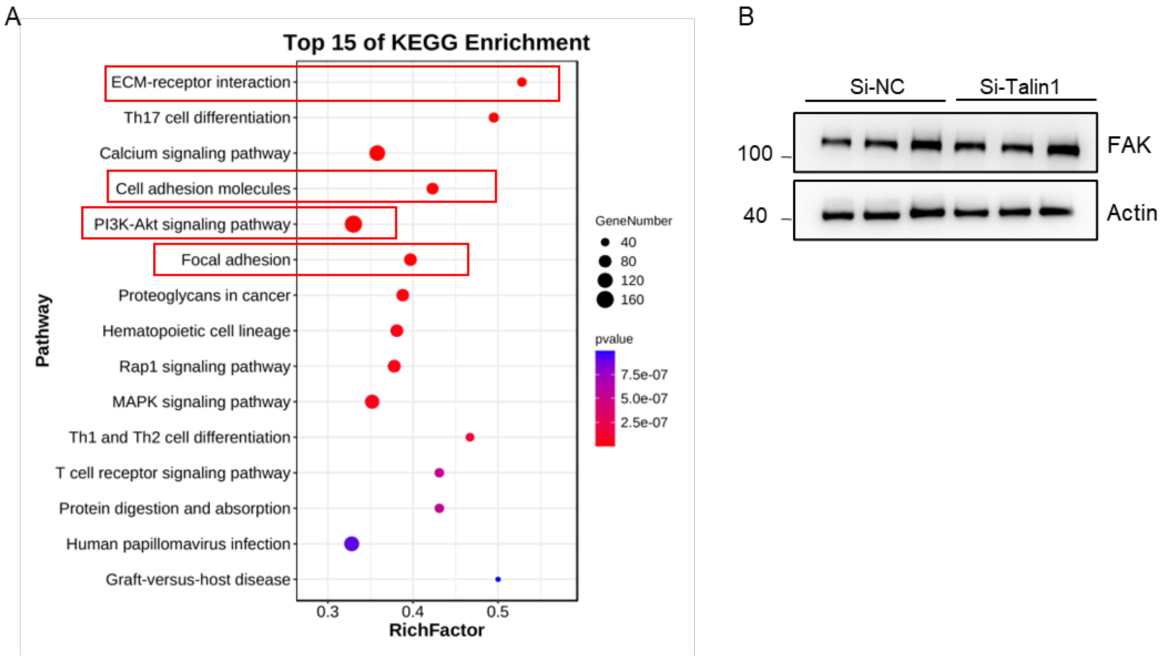

Figure S1. KEGG enrichment. (A) The KEGG enrichment analysis of young and aged mice cortical bone samples. The ECM-receptor interaction, cell adhesion, PI3K-Akt signaling pathway and focal adhesion signaling pathways are highlighted in red boxes. **(B)** Western blot (WB) analysis for FAK protein treated with si-NC (negative control) or si-talin1in MLO-Y4 cells.

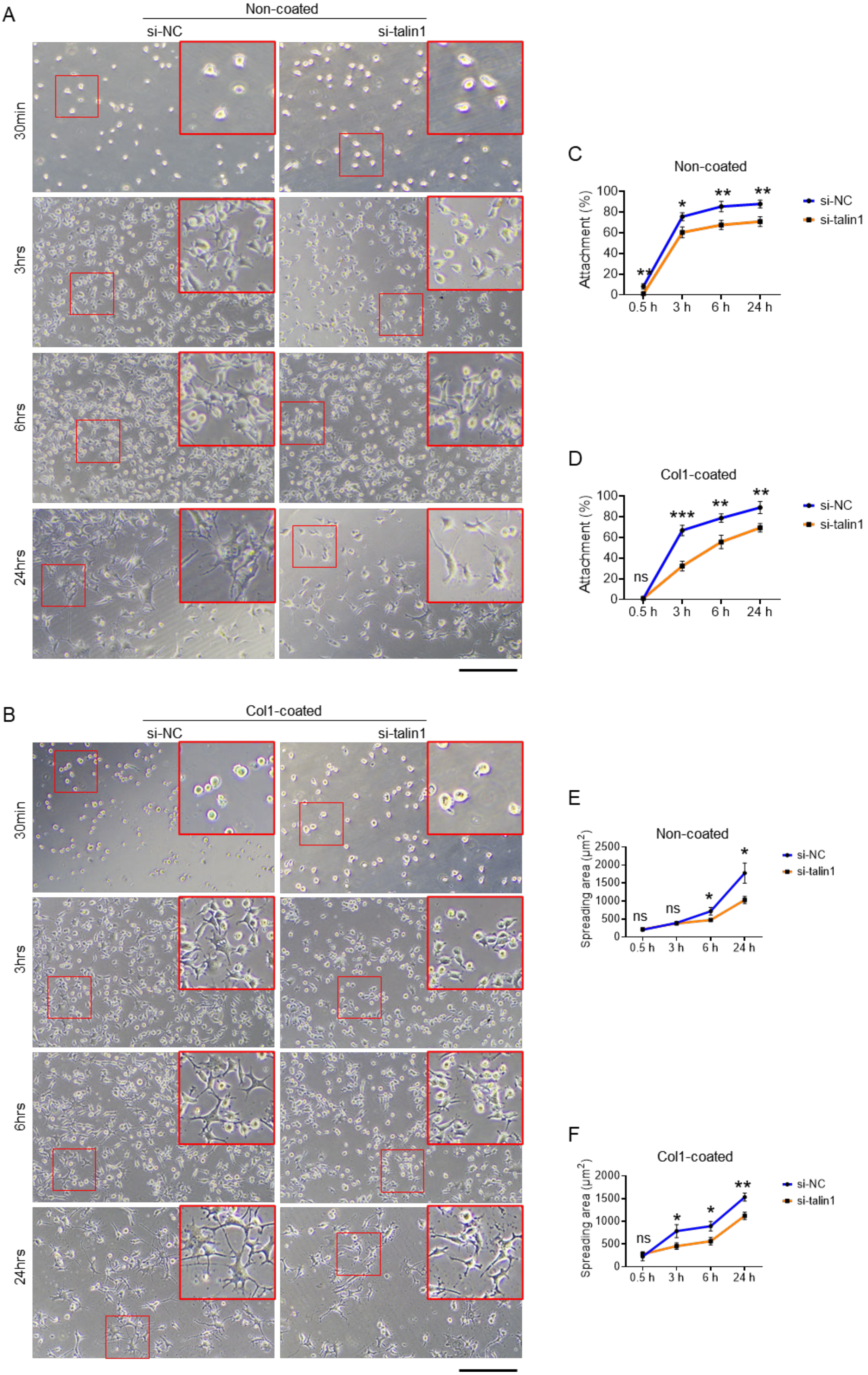
A
Non-coated
si-NC
si-talin1
30min
3hrs
6hrs
24hrs
B
Col1-coated
si-NC
si-talin1
30min
3hrs
6hrs
24hrs
C
Non-coated
Attachment (%)
100
80
60
40
20
0
0.5 h
3 h
6 h
24 h
**
*
**
**
si-NC
si-talin1
D
Col1-coated
Attachment (%)
100
80
60
40
20
0
0.5 h
3 h
6 h
24 h
ns
***
**
**
si-NC
si-talin1
E
Non-coated
Spreading area (μm²)
2500
2000
1500
1000
500
0
0.5 h
3 h
6 h
24 h
ns
ns
*
*
si-NC
si-talin1
F
Col1-coated
Spreading area (μm²)
2000
1500
1000
500
0
0.5 h
3 h
6 h
24 h
ns
*
*
**
si-NC
si-talin1

Figure S2. Osteocyte attachment assay. (A, B) Representative images of si-NC and *si-talin1* MLO-Y4 cells spreading and attachment on non-coated glass surface **(A)** and Col-1 coated surface **(B)** at 0.5, 3, 6, and 24 h after seeding. Scale bar, 100 μm. Statistical analysis of percentage of spreading cells for si-NC and *si-talin1* MLO-Y4 cells on glass surface **(C, E)** and Col-1 coated surface **(D, F)** at 0.5, 3, 6 and 24 h after seeding. All results were expressed as mean ± s.d., n. s. $P > 0.05$, *$P < 0.05$, **$P < 0.01$, ***$P < 0.001$ versus controls, unpaired two-tailed Student's *t* test.

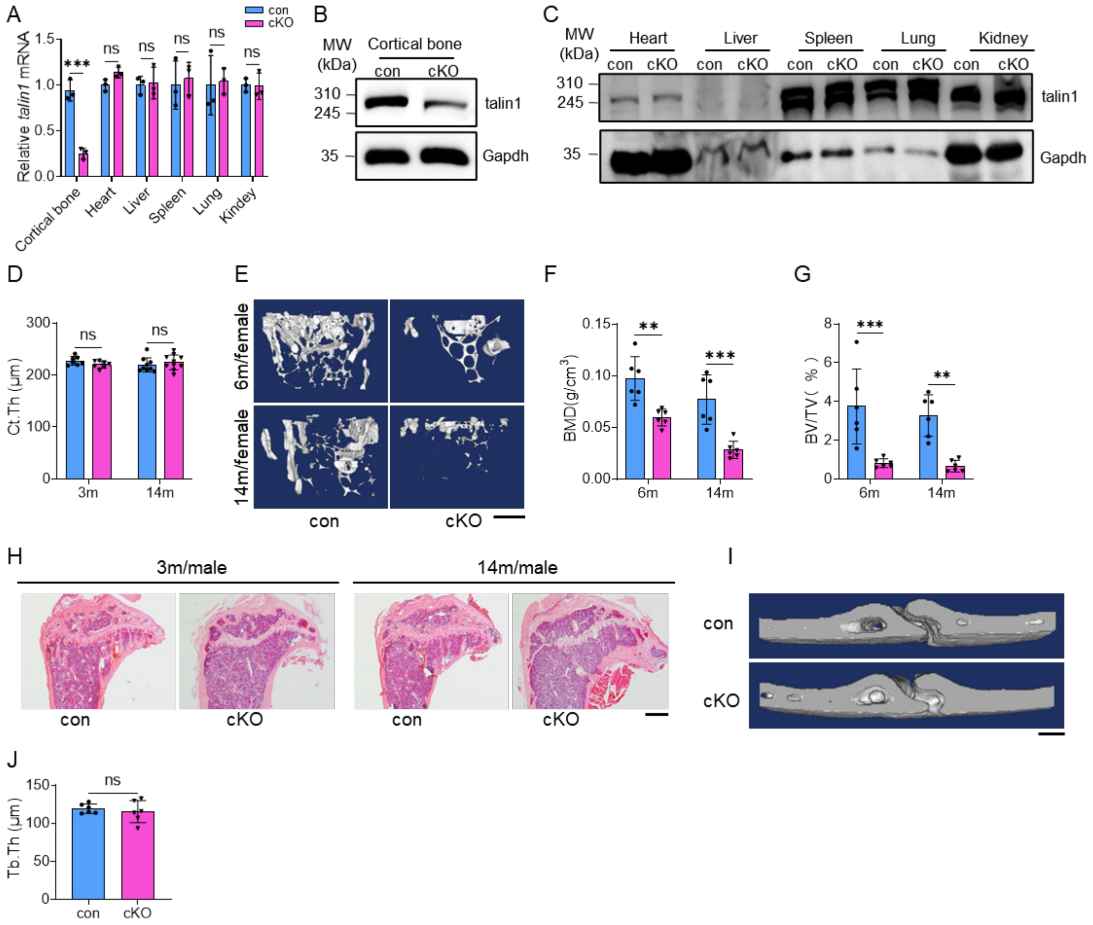

Figure S3. Validation of talin1 loss in conditional knockout mice. **(A)** Real-time RT-PCR (qPCR) analyses of *talin1* expression in different types of tissues isolated from 6-mo-old male control (con) and cKO mice. Data was normalized to *Gapdh* mRNA. N = 3 per group. **(B, C)** Western blot (WB) analysis of talin1 protein expression from the cortical bone and other tissues of 6-mo-old control and cKO mice. Gapdh was used for loading control. **(D)** Quantitative analyses of cortical thickness (Ct.Th) of distal femurs from male con and cKO mice with the indicated ages. N = 7, 3-mo-old male mice; N = 9, 14-mo-old male control mice; N = 10, 14-mo-old male cKO mice. **(E)** 3D reconstruction from µCT scans of tibia from female control and cKO mice. **(F-G)** Quantitative analyses of the bone mineral density (BMD) and bone volume/tissue volume (BV/TV) of distal femurs from female control and cKO mice with the indicated ages. N = 6 per group, biological replicates. **(H)** H/E staining of tibial sections of control and cKO male mice with the indicated ages. Scale bar, 500 µm. **(I)** 3D reconstruction from µCT scans of skull from male control and cKO mice. Scale bars, 1 mm. **(J)** Quantitative analyses of the skull from male control and cKO mice. N = 6 per group,

biological replicates. All results were expressed as mean ± s.d., n. s. $P > 0.05$, $^{**}P < 0.01$, $^{***}P < 0.001$ versus controls, unpaired two-tailed Student's *t* test.

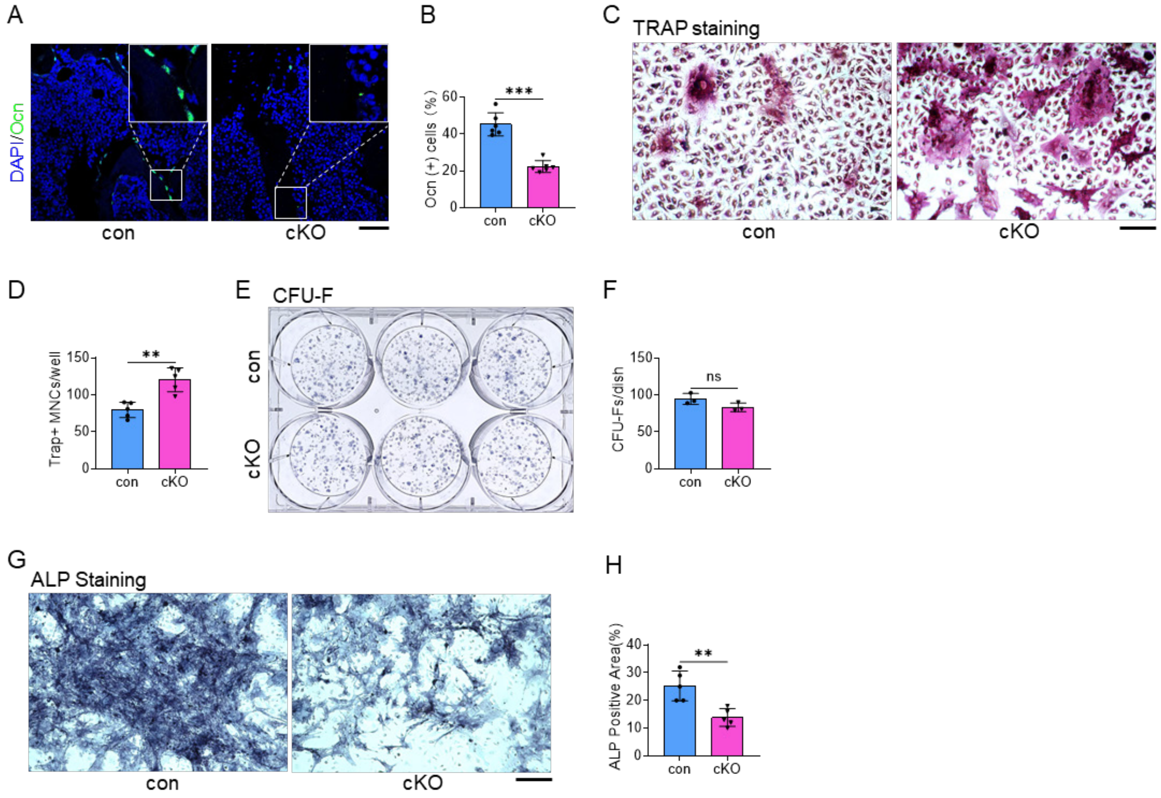

Figure S4. Talin1 loss leads to reduced bone formation and enhanced osteoclast formation in mice. (A) IF staining of osteocalcin (Ocn) from the tibial sections of 3-mo-old control and cKO mice. Scale bar, 50 µm. **(B)** Quantification analysis of osteocalcin-positive osteocytes in A. N = 6 per group, biological replicates. **(C)** TRAP staining of bone marrow monocytes (BMMs) derived from 6-mo-old male con and cKO mice. Scale bar, 100 µm. **(D)** quantitative analyses of $TRAP^{+}$ multinucleated cells in G. N = 5 per group, biological replicates. **(E)** Colony forming unit-fibroblast (CFU-F) assays of bone marrow nucleated cells derived from 6-mo-old male control and cKO mice. **(F)** Quantitative data of C. N = 3 per group, biological replicates. **(G)** ALP staining for in vitro osteoblastic differentiation of the primary BMSCs derived from 6-mo-old male control and cKO mice. **(H)** Quantitative data of E. N = 3 per group, biological replicates. All results were expressed as mean ± s.d., n. s. $P > 0.05$, $^{**}P < 0.01$, $^{***}P < 0.001$ versus controls, unpaired two-tailed Student's *t* test.

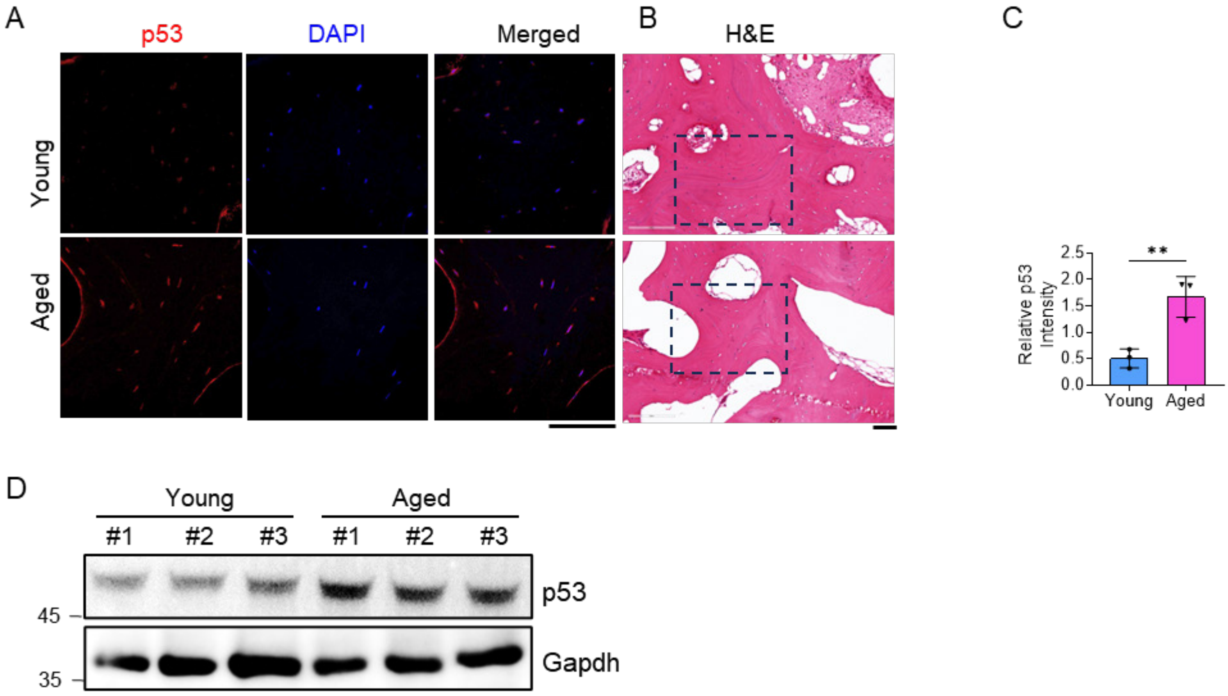

Figure S5. Osteocyte p53 is up-regulated during skeletal aging in humans. (A, B) IF staining ang Hematoxylin and eosin (H/E) staining for p53 expression of young (29 years old) and aged (75 years old) human bone samples. Scale bars, 100 μm. **(C)** Quantification data of B. N = 3, biological replicates. All results were expressed as mean ± s.d., n. s. $**P < 0.01$ versus controls, unpaired two-tailed Student's t test. **(D)** WB analyses for p53 expression of young and aged human bone samples.

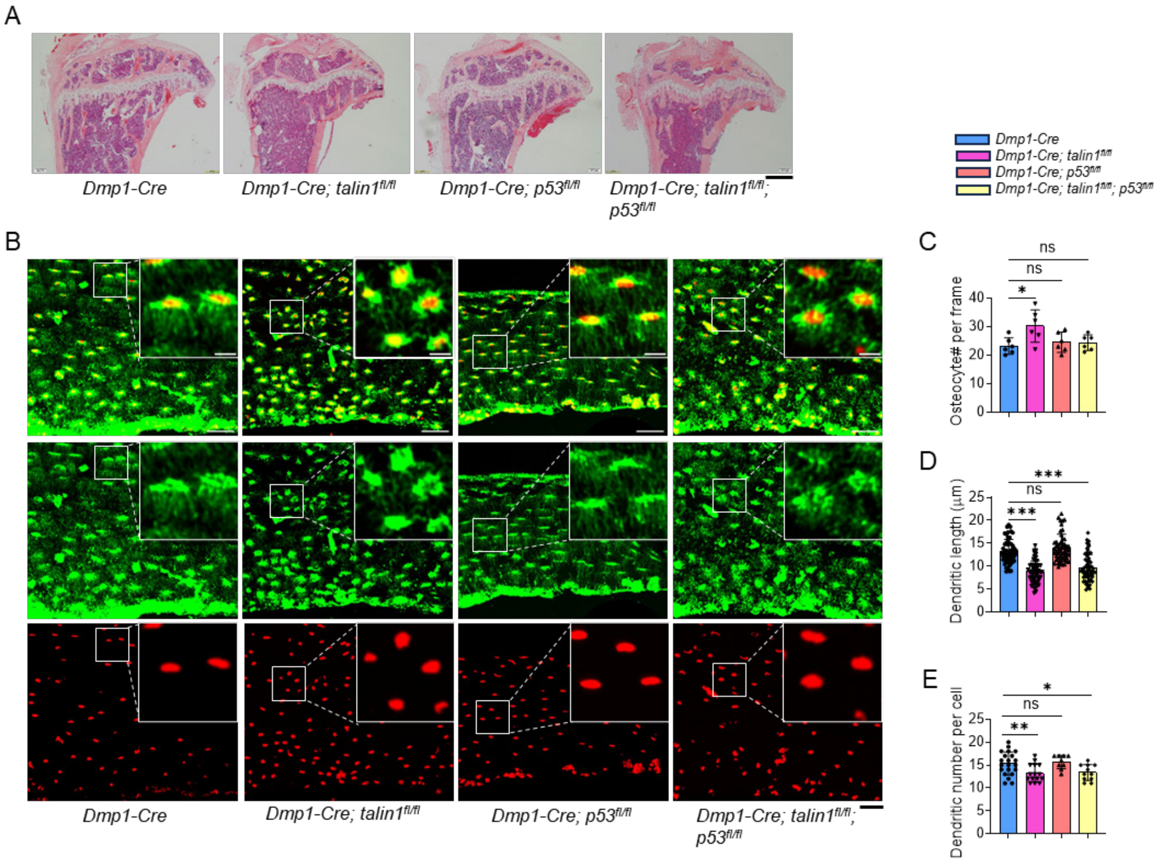

Figure S6. HE and IF staining of tibial sections. (A) H/E staining of tibial sections of 3-mo-old mice of the indicated genotypes. Scale bar, 500 μm. **(B)** F-actin cytoskeleton staining with tibia sections from indicated genotype. Scale bar, 30 μm. **(C-E)** Quantitative analysis of B. N = 6 per group, biological replicates. All results were expressed as mean ± s.d., n. s. P > 0.05, **P* < 0.05, **P < 0.01, ***P < 0.001 versus controls, ordinary one-way ANOVA.
